# Potential False-Positive and False-Negative Results for COVID-19 IgG/IgM Antibody Testing After Heat-Inactivation

**DOI:** 10.3389/fmed.2020.589080

**Published:** 2021-01-18

**Authors:** Jie Lin, Wei Dai, Weiwei Li, Li Xiao, Tao Luo, Yanju Guo, Yang Yang, Ying Han, Peiran Zhu, Qiuyue Wu, Bangshun He, Jian Wu, Xinyi Xia

**Affiliations:** ^1^COVID-19 Research Center, Institute of Laboratory Medicine, Jinling Hospital, Nanjing University School of Medicine, Nanjing Clinical College of Southern Medical University, Nanjing, China; ^2^The 904th Hospital, Wuxi, China; ^3^Joint Expert Group for COVID-19, Department of Laboratory Medicine & Blood Transfusion, Wuhan Huoshenshan Hospital, Wuhan, China; ^4^General Clinical Research Center, Nanjing First Hospital, Nanjing Medical University, Nanjing, China

**Keywords:** COVID-19, SARS-CoV-2, IgG and IgM antibody, heat-inactivation, indirect immunity method

## Abstract

**Objectives:** With the worldwide spread of coronavirus disease 2019 (COVID-19) caused by severe acute respiratory syndrome coronavirus 2 (SARS-CoV-2), various antibody detection kits have been developed to test for SARS-CoV-2– specific IgG, IgM, and total antibody. However, the use of different testing methods under various heat-inactivation conditions might affect the COVID-19 detection results.

**Methods:** Seven different antibody detection kits produced by four manufacturers for detection of SARS-CoV-2 IgG, IgM, and total antibody were tested at Wuhan Huoshenshan Hospital, China. Most of the kits used the indirect immunity, capture, and double-antigen sandwich methods. The effects of various heat-inactivation conditions on SARS-CoV-2-specific IgG, IgM, and total antibody detection were analyzed for the different test methods.

**Results:** Using the indirect immunity method, values for SARS-CoV-2 IgG antibody significantly increased and those for IgM antibody decreased with increasing temperature of heat-inactivation using indirect immunity method. However, values for SARS-CoV-2 IgM and total antibody showed no change when the capture and double-antigen sandwich methods were used. The changes in IgG and IgM antibody values with the indirect immunity method indicated that heat-inactivation could affect COVID-19 detection results obtained using this method. In particular, 18 (22.2%) SARS-CoV-2 IgM positive samples were detected as negative with heat-inactivation at 65°C for 30 min, and one (25%) IgG negative sample was detected as positive after heat-inactivation at 56°C for 60 min and 60°C for 30 min.

**Conclusions:** Heat-inactivation could increase SARS-CoV-2 IgG antibody values, and decrease IgM antibody values, causing potential false-positive or false-negative results for COVID-19 antibody detection using the indirect immunity method. Thus, before conducting antibody testing, the testing platforms should be evaluated in accordance with the relevant requirements to ensure accurate COVID-19 detection results.

## Introduction

Coronavirus disease 2019 (COVID-19), caused by severe acute respiratory syndrome coronavirus 2 (SARS-CoV-2), has rapidly spread worldwide, threatening human health and economic development (1). As of 1 December 2020, the World Health Organization has documented 63,691,642 confirmed cases of COVID-19, with 1,476,277 deaths (2.32%) worldwide. On January 30, 2020, the World Health Organization issued a statement announcing that COVID-19 was a Class I public health emergency of global concern (2).

In the early stages of the COVID-19 outbreak, nucleic acid detection was the main method used for the detection of this disease (3). However, this method required samples from throat swabs, which were highly infectious, and methodological limitations led to long detection periods (4). According to the Diagnosis and Treatment Protocol for Novel Coronavirus Pneumonia (7th Trial Version) in China released on March 3, 2020 (5), serological testing for SARS-CoV-2-specific IgG antibody (Ab) and IgM Ab was identified as suitable for the detection of COVID-19. On April 1, 2020, the US Food and Drug Administration authorized the first SARS-CoV-2 Ab detection kit (6).

Heat-inactivation is a common virus inactivation method used in laboratories. Owing to serious pathogenicity and infectivity of COVID-19, the Inspection Branch of the Chinese Association of Laboratory Medicine required that effective biological safety precautions were taken in laboratories when analyzing the virus; among these precautions, it was recommended that serum samples should be heat-inactivated before serological Ab detection to ensure biosecurity. However, heat-inactivation could affect the values of IgG and IgM Ab detection, with possible effects on the results of clinical tests for COVID-19. At present, at Wuhan Huoshenshan Hospital of China, seven different Ab detection kits for SARS-CoV-2-specific IgG, IgM, and total Ab, produced by four manufacturers, have been used in clinical tests. The current study investigated the effects of various heat-inactivation conditions (including 56°C for 30 min, 56°C for 45 min, 56°C for 60 min, 60°C for 30 min and 65°C for 30 min) on the SARS-CoV-2 antibody detection with the indirect immunity, capture, and double-antigen sandwich methods, using chemiluminescence microparticle immunoassay (CMIA) and up-converting phosphor technology (UPT). Potential false-positive and false-negative rates for COVID-19 detection were also analyzed.

## Materials and Methods

### Data Collection

All serum samples used in this study were collected from patients admitted to Wuhan Huoshenshan Hospital of China between February 4 and April 12, 2020, and diagnosed with COVID-19 infection by nucleic acid testing according to the Diagnosis and Treatment Protocol for Novel Coronavirus Pneumonia (7th Trial Version). Huoshenshan Hospital was established in early February, 2020, and built within 10 days, it is one of the biggest designated hospitals for COVID-19 patients in China, with well-trained clinicians and up-to-date laboratory equipment. This study was approved by the Medical Ethical Committee of Huoshenshan Hospital, Wuhan, China (HSSLL011 and HSSLL012), and written informed consent was obtained from the patients.

### Experimental Regents and Instruments

Seven different SARS-CoV-2-specific IgG, IgM, and total Ab detection kits, using the indirect immune method, capture method, or double-antigen sandwich method, based on CMIA and UPT, and produced by four different manufacturers, were tested at Wuhan Huoshenshan Hospital of China (Table 1). In which, the SARS-CoV-2-specific IgG and IgM Ab detection kits, using the indirect immune method, based on CMIA, produced by the same manufacture were under the approval process by National Medical Products Administration of China. The other five different Ab detection kits, including SARS-CoV-2-specific IgG, IgM, and total antibody detection kits, using the indirect immune method, capture method, or double-antigen sandwich method, based on CMIA and UPT, produced by three manufactures had been approved by National Medical Products Administration of China, and the corresponding approval number of National medical device products had been released online. A UPT immunoassay analyzer (UPT-3A-1200), automatic chemiluminescence immunoassay analyzer (Caris200), automatic chemiluminescence immunoassay analyzer (CL-2000i), and automatic chemiluminescence immunoassay analyzer (iFlash 3000-H) were used in this study.

**Table 1:** SARS-CoV-2 antibody detection kits.

**Manufacturer**	**A**		**B**		**C**		**D**
Antibody	IgG Ab	IgM Ab	IgG Ab	IgM Ab	IgM Ab	Total Ab	Total Ab
Method	Indirect immunity		Indirect immunity		Capture	Double-antigen	Double-antigen
Technology	Chemiluminescence microparticle immunoassay		Chemiluminescence microparticle immunoassay		Chemiluminescence microparticle immunoassay		Up-converting phosphor technology

### Experimental Methods

**Figure 1:**
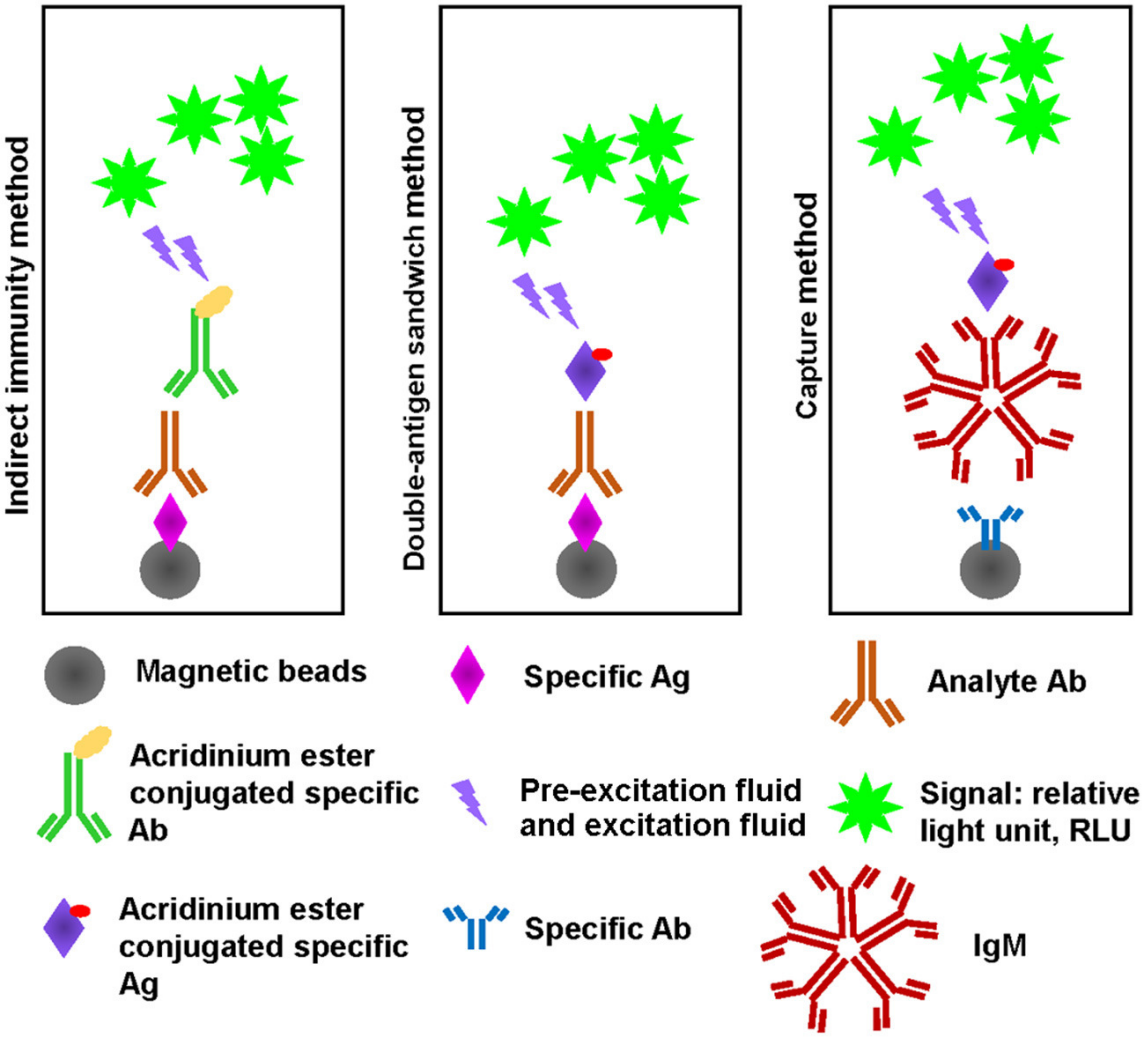
Schematic diagram of indirect immunity, double-antigen sandwish, and capture method.

Owing to the specificity of Wuhan Huoshenshan Hospital, detection SARS-CoV-2 Ab without heat-inactivation was used as a control. Serological tests for, and total Ab were performed using the corresponding Ab detection kits, according to the manufacturers' instructions. In brief, for SARS-CoV-2-specific IgG and IgM Ab detection using indirect immunity method, magnetic beads coated with the specific IgG or IgM antigen (Ag) were mixed with analyte serum samples to form Ag/Ab complex, after washing, second Ab Mouse anti-human IgG or IgM coated with acridinium ester were mixed to form Ag/Ab/second Ab complex, after washing, pre-excitation fluid and excitation fluid was added, then the relative light unit (RLU) of signal was detected. For SARS-CoV-2 total Ab detection using double-antigen sandwich method, were mixed with analyte serum samples to form Ag/Ab complex, after washing, specific Ag coated with acridinium ester were mixed to form Ag/Ab/Ag complex, after washing, pre-excitation fluid and excitation fluid was added, then the RLU of signal was detected. For SARS-CoV-2-specific IgM Ab detection using capture method, magnetic beads coated with anti-human specific IgM Ab mixed with analyte serum samples, after washing, specific Ag coated with acridinium ester were mixed to form Ab/IgM/Ab complex, after washing, pre-excitation fluid and excitation fluid was added, then the RLU of signal was detected (Figure 1). Before testing, serum samples were heat-inactivated in a water bath at 56°C for 30 min, 56°C for 45 min, 56°C for 60 min, 60°C for 30 min, and 65°C for 30 min.

### Statistical Analysis

Data are presented as mean ± standard deviation (SD), and measured by GraphPad Prism 8.0. Statistical significance was analyzed by two-tailed paired student's *t*-test. Differences at *p* < 0.05 were considered to statistical significance.

## Results

### Effects of Heat-Inactivation Conditions on Indirect Immunity Method

**Figure 2:**
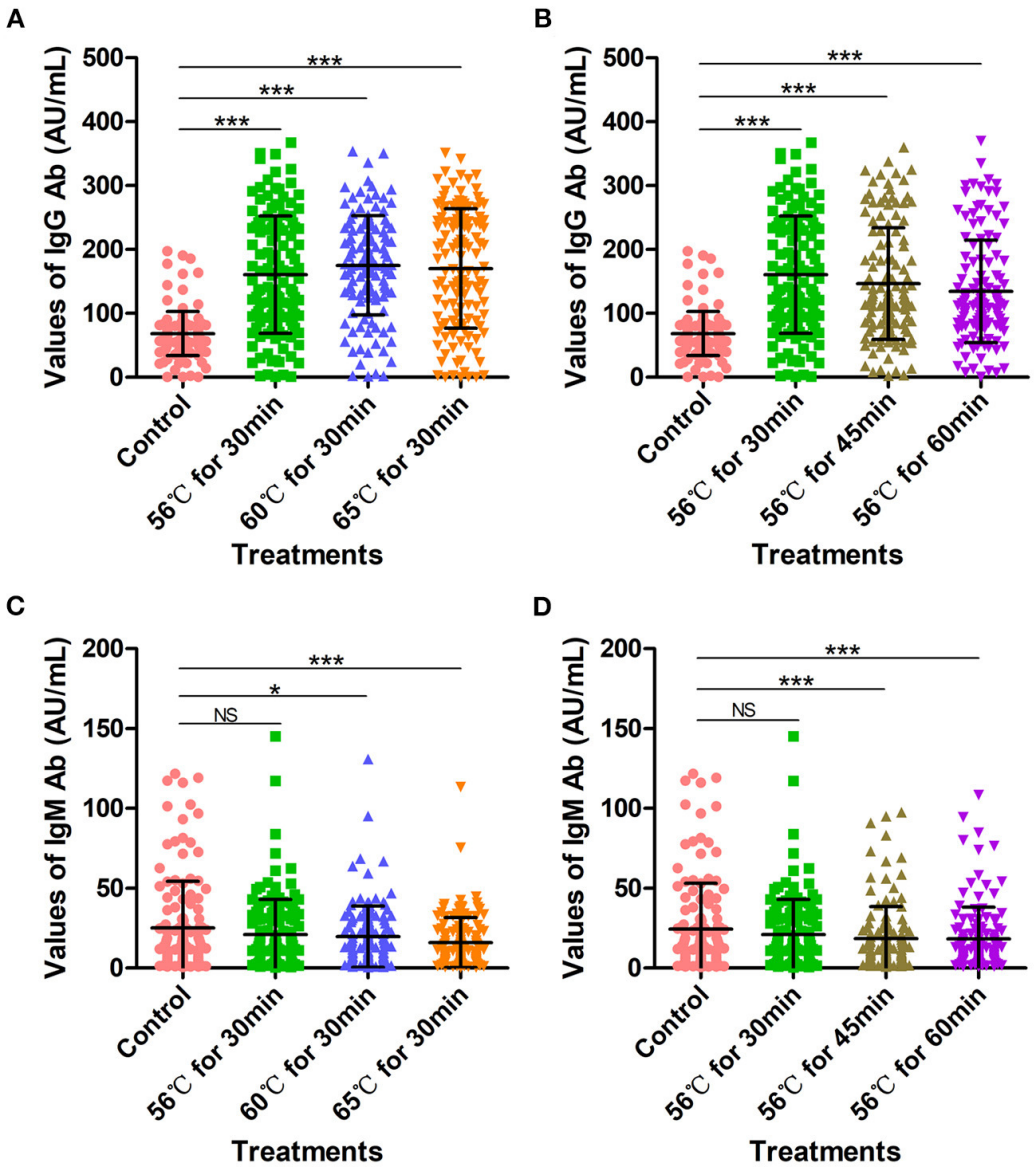
SARS-CoV-2-specific IgG and IgM antibody detection values with indirect immunity-based kit produced by manufacturer A. **(A)** SARS-CoV-2 IgG antibody detection values after heat-inactivation for 30 min. Before testing, a total of 129 samples were heat-inactivated at 56, 60, or 65°C for 30 min. **(B)** SARS-CoV-2 IgG antibody detection values after heat-inactivation at 56°C. Before testing, a total of 129 samples were heat-inactivated at 56°C for 30, 45, or 60 min. **(C)** SARS-CoV-2 IgM antibody detection values after heat-inactivation for 30 min. Before testing, a total of 129 samples were heat-inactivated at 56, 60, or 65°C for 30 min. **(D)** SARS-CoV-2 IgM antibody values after heat-inactivation at 56°C. Before testing, a total of 129 samples were heat-inactivated at 56°C for 30, 45, or 60 min. The detection of SARS-CoV-2 antibody without heat-inactivation were used as control. NS, non-significant; *p ≤ 0.05; ***p ≤ 0.001.

A total of 129 serum samples collected from COVID-19 patients admitted to Wuhan Huoshenshan Hospital were tested with SARS-CoV-2 specific IgG and IgM Ab detection kits using the indirect immunity method, produced by manufacturer A. Before testing, samples were heat-inactivated in water bath at 56°C for 30 min, 56°C for 45 min, 56°C for 60 min, 60°C for 30 min, or 65°C for 30 min. The average IgG Ab value for the control group without heat-inactivation was 68.46 AU/mL, whereas those obtained after heat-inactivation at 56°C for 30 min, 60°C for 30 min, and 65°C for 30 min were significantly higher (*p* < 0.001) at 160.44, 175.21, and 170.21 AU/mL, respectively (Figure 2A). In addition, when serum samples were heat-inactivated at 56°C, the IgG Ab values after heat-inactivation for 30, 45, and 60 min were significantly higher (*p* < 0.001) than control values, with averages of 160.44, 146.61, and 134.37 AU/mL, respectively (Figure 2B, Supplementary Table 1).

The average IgM Ab value in the control group was 24.35 AU/mL; for heat-inactivation time of 30 min, IgM Ab values decreased compared with controls as the temperature of heat-inactivation increased (*p* < 0.05). In particular, for heat-inactivation at 65°C, IgM Ab levels were very significantly decreased compared with controls (*p* < 0.001). The average IgM Ab values obtained after heat-inactivation at 56°C for 30 min, 60°C for 30 min, and 65°C for 30 min were 20.95 AU/mL, 19.70 AU/mL, and 15.98 AU/mL, respectively (Figure 2C). Notably, even at 56°C, heat-inactivation for 30 min, 45 min, and 60 min led to lower IgM Ab values compared with controls (*p* < 0.05), with average values of 20.95, 18.49, and 18.22, respectively (Figure 2D, Supplementary Table 2).

These increases in SARS-CoV-2-specific IgG Ab values and decreases in IgM values obtained with the indirect immunity method after heat-inactivation could cause potential false-positive and false-negative results in COVID-19 detection. As shown in Table 2, one (25%) IgG Ab-negative sample was determined as positive owing to increased IgG values after heat-inactivation at 56°C for 60 min and 60°C for 30 min (Table 2). Correspondingly, a total of 12 (16.2%), 10 (13.5%), 18 (24.3%), 12 (16.0%) and 13 (17.6%) IgM-positive samples were detected as negative, owing to IgM values decreasing after heat-inactivation at 56°C for 30 min, 60°C for 30 min, 65°C for 30 min, 56°C for 45 min, and 56°C for 60 min, respectively (Table 2).

**Table 2:** Potential false-positive and false-negative rates after heat-inactivation for SARS-CoV-2 IgG and IgM Ab detection with indirect immunity-based kit produced by manufacturer A.

**IgG Ab**		**56**^****°****^**C for 30 min**		**Total**	**56**^****°****^**C for 45 min**		**Total**	**56**^****°****^**C for 60 min**		**Total**	**60**^****°****^**C for 30 min**		**Total**	**65**^****°****^**C for 30 min**		**Total**
		**Po**	**Ne**		**Po**	**Ne**		**Po**	**Ne**		**Po**	**Ne**		**Po**	**Ne**	
Control	Po	125 (100.0%)	**0 (0.0%)**	125	125 (100.0%)	**0 (0.0%)**	125	125 (100.0%)	**0 (0.0%)**	125	124 (99.2%)	**1 (0.8%)**	125	121 (96.8%)	**4 (3.2%)**	125
	Ne	vitchu**0 (0.0%)**	4 (100.0%)	4	vitchu**0 (0.0%)**	4 (100%)	4	vitchu**1 (25.0%)**	3 (75.0%)	4	vitchu**1 (25.0%)**	3 (75.0%)	4	vitchu**0 (0.0%)**	4 (100.0%)	4
Total		125	4	**129**	125	4	**129**	126	3	**129**	125	4	**129**	**121**	8	**129**
IgM Ab		**Po**	**Ne**	**Total**	**Po**	**Ne**	**Total**	**Po**	**Ne**	**Total**	**Po**	**Ne**	**Total**	**Po**	**Ne**	**Total**
Control	Po	62 (83.8%)	**12 (16.2%)**	74	62 (84.0%)	**12 (16.0%)**	74	61 (82.4%)	**13 (17.6%)**	74	64 (86.5%)	**10 (13.5%)**	74	56 (75.7%)	**18 (24.3%)**	74
	Ne	**16 (29.1%)**	39 (70.9%)	55	**11 (21.4%)**	44 (78.6%)	55	**12 (21.8%)**	43 (78.2%)	55	**17 (30.9%)**	38 (69.1%)	55	**16 (29.1%)**	39 (70.9%)	55
Total		78	51	**129**	73	56	**129**	**73**	56	**129**	81	48	**129**	72	57	**129**

*blue bold, false-negative; red bold, false-positive; Po, positive; Ne, negative*.

**Figure 3:**
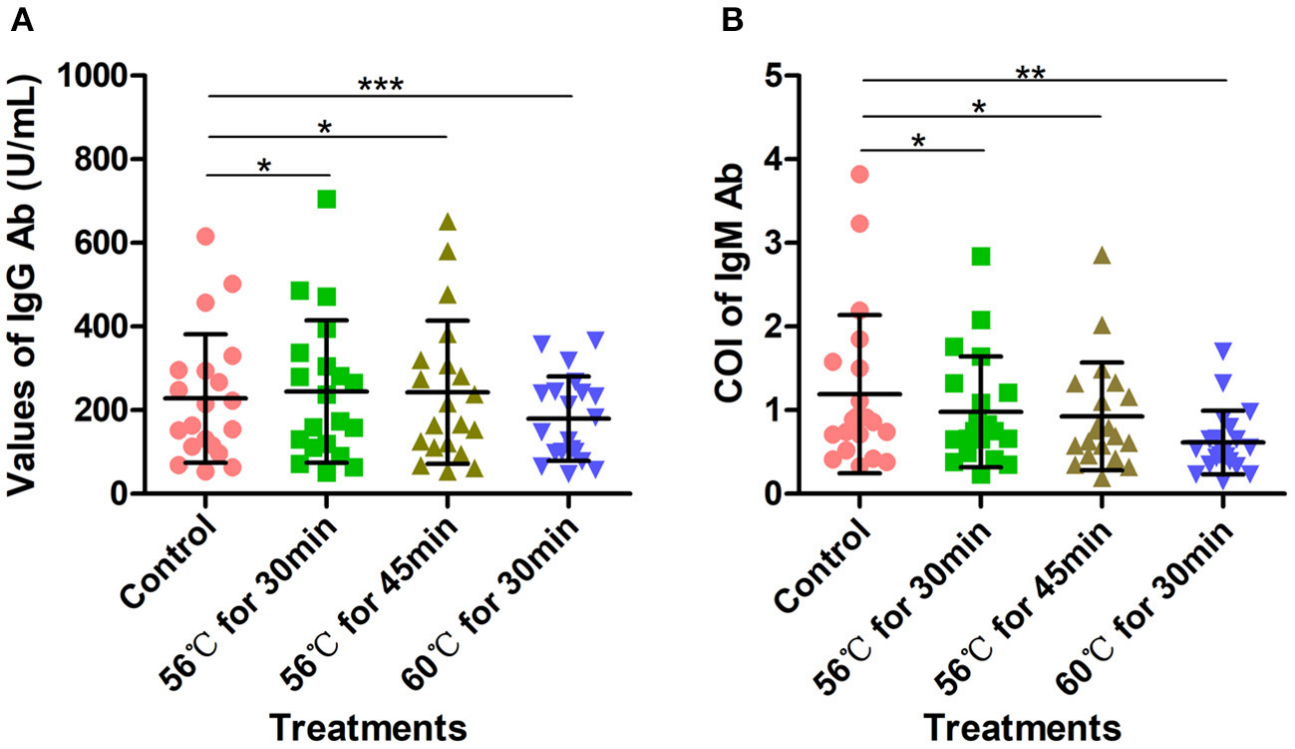
Detection of SARS-CoV-2-specific IgG and IgM antibodies with indirect immunity-based kit produced by manufacturer B. **(A)** Density of SARS-CoV-2-specific IgG antibody detection. Before testing, 20 samples were heat-inactivated at 56°C for 30 min, 56°C for 45 min, or 60°C for 30 min, respectively. **(B)** COI values for SARS-CoV-2-specific IgM antibody. Before testing, 20 samples were heat-inactivated at 56°C for 30 min, 56°C for 45 min, or 60°C for 30 min. The detection of SARS-CoV-2 antibody without heat-inactivation were used as control. *p ≤ 0.05; **p ≤ 0.01; ***p ≤ 0.001.

Another 20 serum samples collected from COVID-19 patients admitted to Wuhan Huoshenshan Hospital were tested with SARS-CoV-2 specific IgG and IgM Ab detection kits produced by manufacturer B, also based on the indirect immunity method. Before testing, samples were heat-inactivated at 56°C for 30 min, 56°C for 45 min, or 60°C for 30 min in a water bath. The IgG Ab values obtained after heat-inactivation at 56°C for 30 min and 56°C for 45 min were significantly higher than those of the control group without heat-inactivation (*p* < 0.05); the average value for the control group was 228.04 U/mL, whereas values of 244.58 U/mL and 242.59 U/mL were obtained after heat-inactivation at 56°C for 30 min and 56°C for 45 min, respectively (Figure 3A). Notably, when samples were heat-inactivated at 60°C for 30 min, the IgG Ab value was significantly decreased in comparison with the control group (*p* < 0.0001), with an average IgG Ab value of 179.55 U/mL, This might have been because the IgG Ab was deactivated by heat-inactivation at a high temperature (Supplementary Table 3). In addition, whereas the control group had an average cutoff index (COI) value of 1.19, heat-inactivation at higher temperature led to lower COI values for IgM Ab; in particular, with heat-inactivation at 60°C for 30 min, IgM Ab values were significantly decreased (*p* < 0.01), with an average COI value of 0.61. For heat-inactivation at a given temperature, IgM Ab values decreased with increasing time of heat-inactivation (*p* < 0.01). The average COI values for IgM Ab with heat-inactivation at 56°C for 30 min and 56°C for 45 min were 0.98 and 0.93, respectively (Figure 3B, Supplementary Table 4).

As shown in Supplementary Table 4, although SARS-CoV-2 IgG Ab values increased after heat-inactivation using the indirect immunity method, all the samples used in this experiment were initially IgG positive; thus, there was no potential for false-positives (Table 3). However, owing to the decreases in SARS-CoV-2-specific IgM Ab values after heat-inactivation, there were five (71.4%) initially IgM-positive samples were determined as negative after heat-inactivation at 60°C for 30 min (Table 3). These results show that heat-inactivation could lead to increased SARS-CoV-2 IgG Ab values and decreased IgM antibody, directly causing false-negative or false-positive results in COVID-19 detection using the indirect immunity method.

**Table 3:** Potential false-positive and false-negative rates after heat-inactivation for SARS-CoV-2 IgG and IgM Ab detection with indirect immunity-based kit produced by manufacturer B.

**IgG Ab**		**56**^****°****^**C for 30 min**		**Total**	**56**^****°****^**C for 45 min**		**Total**	**60**^****°****^**C for 30 min**		**Total**
		**Po**	**Ne**		**Po**	**Ne**		**Po**	**Ne**	
Control	Po	20 (100.0%)	**0 (0.0%)**	20	20 (100.0%)	**0 (0.0%)**	20	20 (100.0%)	**0 (0.0%)**	20
	Ne	**0 (0.0%)**	0 (100.0%)	0	**0 (0.0%)**	0 (100.0%)	0	**0 (0.0%)**	0 (100.0%)	0
Total		20	0	**20**	20	0	**20**	20	0	**20**
IgM Ab		**Po**	**Ne**	**Total**	**Po**	**Ne**	**Total**	**Po**	**Ne**	**Total**
Control	Po	7 (100.0%)	**0 (0.0%)**	7	7 (100.0%)	**0 (0.0%)**	7	2 (28.6%)	**5 (71.4%)**	7
	Ne	**0 (0.0%)**	13 (100.0%)	13	**0 (0.0%)**	13 (100.0%)	13	**0 (0.0%)**	13 (100.0%)	13
Total		7	13	**20**	7	13	**20**	2	18	**20**

*blue bold, false-negative; red bold, false-positive; Po, positive; Ne, negative*.

### Effects of Heat-Inactivation Conditions With Capture Method

**Figure 4:**
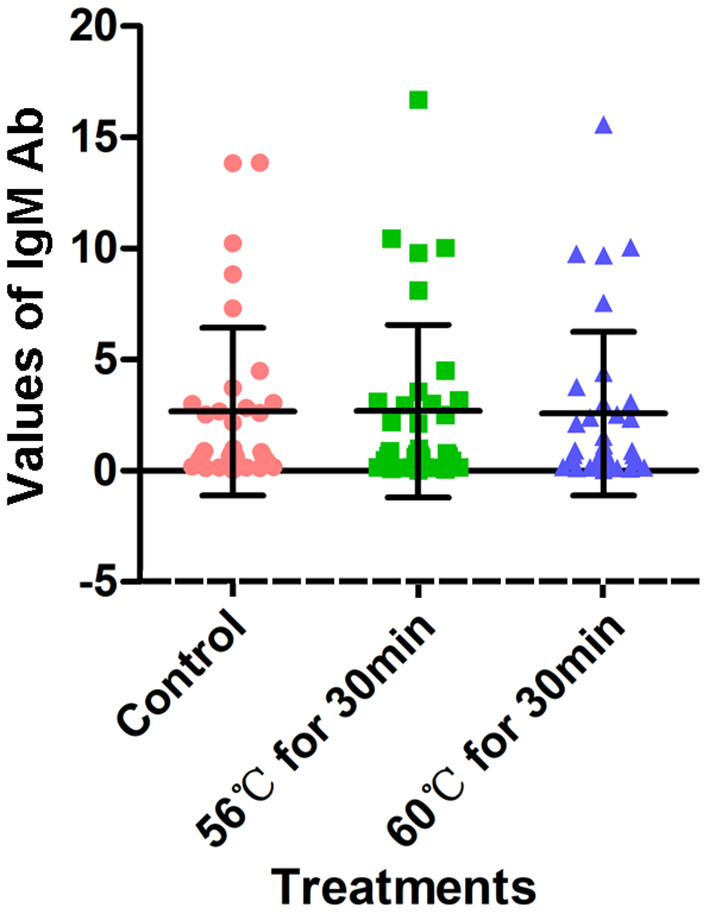
Detection results of SARS-CoV-2-specific IgM antibody with capture method. Before testing, a total of 34 samples were heat-inactivated at 56°C for 30 min or 60°C for 30 min. The detection of SARS-CoV-2 antibody without heat-inactivation were used as control.

A total of 34 serum samples collected from COVID-19 patients admitted to Wuhan Huoshenshan Hospital were tested with a SARS-CoV-2-specific IgM Ab detection kit based on the capture method, produced by manufacturer C. Before testing, serum samples were heat-inactivated at 56°C for 30 min or 60°C for 30 min in a water bath. The detection results for IgM Ab were not affected by heat-inactivation (*p* > 0.05); the average IgM Ab for the control group without heat-inactivation was 2.66, compared with 2.68, and 2.57 after heat-inactivation at 56°C for 30 min and 60°C for 30 min, respectively (Figure 4, Supplementary Table 5). As the different heat-inactivation conditions had no effect on the detection results of IgM Ab with the capture method, there was no effect on COVID-19 detection.

### Effects of Heat-Inactivation Conditions With Double Antigen Sandwich Method

**Figure 5:**
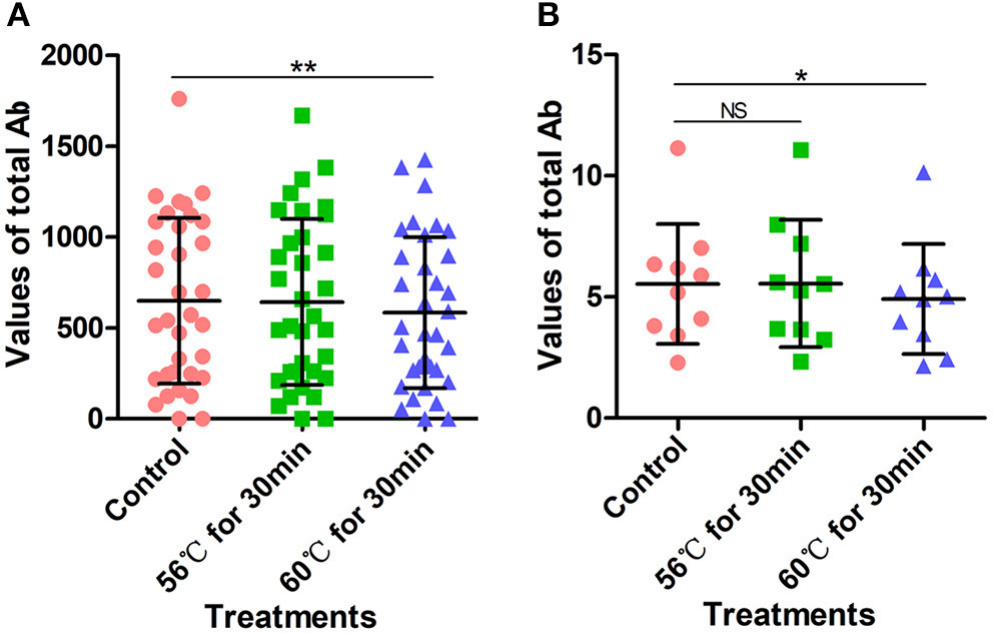
SARS-CoV-2 total antibody values tested using double-antigen sandwich method. **(A)** SARS-CoV-2 total antibody values tested using CMIA kit produced by manufacturer C. Before testing, a total of 34 samples were heat-inactivated at 56°C for 30 min or 60°C for 30 min. **(B)** SARS-CoV-2 total antibody tested using UPT-kit produced by manufacturer D. Before testing, 10 samples were heat-inactivated at 56°C for 30 min or 60°C for 30 min. The detection of SARS-CoV-2 antibody without heat-inactivation were used as control. NS, non-significant; *p ≤ 0.05, **p ≤ 0.01.

The above 34 serum samples were also tested with a SARS-CoV-2 total Ab detection kit based on the double-antigen sandwich method. Before testing, serum samples were heat-inactivated at 56°C for 30 min or 60°C for 30 min in a water bath. Heat-inactivation at 56°C for 30 min had no significant effect on the detection of SARS-CoV-2 total Ab, with an average value of 642.9 compared with 649.76 for the control group (*p* > 0.05). However, the SARS-CoV-2 total Ab values after heat-inactivation at 60°C for 30 min were significantly lower than those of the control group (*p* < 0.0001), with an average value of 584.18 (Figure 5A, Supplementary Table 6). This might have been because of the deactivation of the Ab at the higher temperature. However, although SARS-CoV-2 total Ab values decreased after heat-inactivation at 60°C for 30 min, there was no corresponding effect on COVID-19 detection.

Similarly, 10 serum samples collected from COVID-19 patients admitted to Wuhan Huoshenshan Hospital were tested using a SARS-CoV-2 total Ab detection kit based on the double-antigen sandwich method produced by manufacturer D. Before testing, samples were heat-inactivated at 56°C for 30 min or 60°C for 30 min in a water bath. The average SARS-CoV-2 total Ab value after heat-inactivation at 56°C for 30 min was 5.55, similar to average value of 5.54 for the control group (*p* < 0.05). However, heat-inactivation for the same time of 30 min but at 60°C caused the average total Ab value to decrease to 4.92, which was significantly lower than the control value (*p* < 0.05) (Figure 5B, Supplementary Table 7). Again, this could be attributed to deactivation of the Ab at the higher temperature. In addition, although the SARS-CoV-2 total Ab value decreased after heat-inactivation at 60°C for 30 min, there was no effect on the detection of COVID-19 with the double-antigen sandwich method.

## Discussion

COVID-19 is a highly infectious disease, with an R_0_ value of 3.0–3.5 (7). Early prevention, identification, and diagnosis are particularly important to control the spread of this disease (8). Although the nucleic acid test is the gold standard for COVID-19 detection, it has a long detection time, and the results are susceptible to various factors including the quality of the specimen, the site of viral infection, and the amount of viral expression (9). Serological Ab detection has been added to the Diagnosis and Treatment Protocol for Novel Coronavirus Pneumonia (7th Trial Version) in China, as an auxiliary method for COVID-19 detection, and can be used in conjunction with nucleic acid detection to achieve fast screening for COVID-19 (10).

CMIA is a novel analysis method, takes full advantage of the rapid automation of magnetic separation, the high sensitivity of chemiluminescence, and the specificity of immunoassays. Commonly used of CMIA methods include the indirect immunity method, capture method, solid-phase antigen competition method, and double-antibody sandwich method. The specificity of the reaction between antigen and antibody is determined by the spatial configuration of the antigenic determinant and the antigen-binding region of the Ab (11). Notably, similar to globulins, the resistance to physical and chemical factors of IgG and IgM Ab could be destroyed by heating to 60–70°C; various enzymes and substances that cause protein coagulation and denaturation also lead to loss of function of Abs (12).

In this study, the indirect immunity, capture, and double-antigen sandwich methods were used to detect SARS-CoV-2-specific IgG, IgM, and total Ab. The indirect immunity method has high sensitivity and economical, as fewer labeled Abs are needed and more than one labeled secondary Ab can bind to the primary Ab (13). However, its disadvantages include the possibility of cross-reactivity of the secondary Ab with the adsorbed antigen, which could increase background noise. The capture method, also known as the reverse indirect method, is mainly used for the determination of certain Ab subtype components such as IgM in serum. Owing to the co-existence in serum of IgM and IgG specific to a certain antigen, the presence of IgG can interfere with the measurement of IgM. Therefore, a secondary Ab against IgM is first attached to a solid phase carrier to capture all IgM in the sample, and an antigen specific to IgM to be tested is added. This method has higher stability compared with the indirect immunity method and commonly used for IgM Ab detection. However, the disadvantages of the capture method include its relatively low sensitivity, which decreases its range of applications.

In order to improve biological safety, infectious materials and serum samples are required to be heat-inactivated by reliable methods before serological testing (14). In a previously published investigation of heat-inactivation stability of SARS (15), Rabenau et al. found that the titer of SARS virus was lower than the detection limit after being heat-inactivated at 56°C for 30 min; after heat-inactivation at 60°C for 30 min, there was no residue of SARS virus (16). Kariwa et al. also found no infectious SARS virus after heat-inactivation at 56°C for 60 min or longer (17). However, heat-inactivation of the virus might have an impact on the spatial conformation and protein structure of the antigen-Ab binding region, possibly leading false-positive and false-negative results (14).

In this study, the serological detection of SARS-CoV-2-specific IgG Ab without heat-inactivation was compared with detection after heat-inactivation at 56°C for 30 min, 45 min, and 60 min, and at 60 or 65°C for 30 min, using the indirect immunity method. As the time and temperature of heat-inactivation increased, IgG Ab values significantly increased. When different temperatures of heat-inactivation were compared, IgG Ab values increased after heat-inactivation at 56°C, and increased further after heat-inactivation at 60°C, whereas they decreased after heat-inactivation at 65°C. This phenomenon might occur because the structure of IgG is composed of two heavy and two light chains linked together, creating a large monomeric molecules with a tetrameric quaternary structure, formed by the polymerization of itself under certain temperature conditions. Under the condition of heat-inactivation treatment, the monomeric molecules of IgG antibody could be aggregated, hence the detection values of IgG antibody increase. It has been reported that the thermal polymerization conditions of IgG directly affect the structure of its products and its biological reactivity. For a polymerization time of 30 min, thermal polymerization of IgG occurs in a narrow temperature range of 60–64°C. However, as the thermal polymerization temperature increases, its degree of polymerization and the products increase, as dose its binding capacity (18). The binding capacity is strongest at a thermal polymerization temperature of 62°C, and it can react with the epitope on the Fc segment of IgG molecules (19). During thermal polymerization, the conformation of IgG changes, and the number and accessibility of active sites increase, reaching maximum values under certain conditions. As the thermal polymerization conditions are strengthened, the reaction activity of the product begin to decline. This might because the further increase in the degree of polymerization reduces the accessibility of the reaction site, or because the severe denaturation conditions cause the destruction of the original active site. In addition, excessively high temperatures can cause protein denaturation. Although this IgG aggregation is antigen-nonspecific, and the enhanced signal has no relationship to the recognition of viral antigens, increasing IgG Ab could cause potential false-positive results in COVID-19 detection.

In this study, the serological detection of SARS-CoV-2-specific IgM Ab before heat-inactivation was compared with detection after heat-inactivation at 56°C for 30 min, 56°C for 45 min, 56°C for 60 min, 60°C for 30 min, and 60°C for 30 min. The results indicated that IgM Ab values decreased with increasing time and temperature of heat-inactivation when using the indirect immunity method, possibly because the stability of IgM Ab is much lower than that of IgG Ab (20). It was known that IgM is a symmetrical pentamer structure, where all heavy chains and all light chains are identical. Although the large size (900 kDa) of IgM, its structure could be denatured by the heat-inactivation treatment. In clinical tests, heat-inactivation is a common method to reduce the impact of IgM. The IgM pentamer is an asymmetrical pentagon with an open groove that can bind to specific proteins (21). Heat-inactivation could destroy the polygonal structure of IgM, thereby affecting the specific binding of antigen to Ab (22). There is also evidence that heating could cause false-negative results in the detection of IgM. This is consistent with some of the results of this study.

Additionally, proteins have evolved to have disulfide bonds in their natural conformations, which contribute to thermodynamic stability. These disulfide bonds are broken during heating, and the protein undergoes irreversible denaturation through the disulfide-thiol exchange reaction. Methanethiosulphonate (MTS) could specifically suppress the heat-induced disulphide-thiol exchange reaction, and improve the heat-resistance of proteins. Combining MTS reagents with glycinamide, further enhanced protein stabilization (23). This aspect was not investigated in the current study, but it should be the subject of further research. Different manufacturers select different specific binding sites of antigens, and the various methods differ in their products. The SARS-CoV-2-specific IgG antibody detection kits produced by manufactures B were certainly added with MTS. In addition, heating changes the composition and structure of serum proteins, which affects matrix effects in serum detection results.

Currently, the testing process for COVID-19 is not standardized; there is an urgent need to reduce the development time of detection kits and test platforms, and there has not been sufficient evaluation and clinical verification using large samples. Therefore, factors that might cause inaccurate results need to be considered. Before conducting tests, the testing methods and platforms should be evaluated in accordance with the relevant requirements, in order to reduce potential false-negative and false-positive results, and provide accurate results for COVID-19 detection.

## Data Availability Statement

The raw data supporting the conclusions of this article will be made available by the authors, without undue reservation.

## Ethics Statement

The study has been reviewed and approved by the Medical Ethical Committee of Huoshenshan Hospital, Wuhan, China (HSSLL011 and HSSLL012). Written informed consent was obtained from the individual(s) for the publication of any potentially identifiable images or data included in this article.

## Author Contributions

XX and JW conceived the study. JL performed the experiments, collected data, and wrote the manuscript. WD, WL, and LX analyzed the data, generated the figures, and editor the manuscript. TL, YG, YY, and YH contributed to the data analysis. PZ, QW, and BH contributed to the manuscript editor. All authors contributed to the article and approved the submitted version.

## Conflict of Interest

The authors declare that the research was conducted in the absence of any commercial or financial relationships that could be construed as a potential conflict of interest.
