# Supplementary material for: Potential False-Positive and False-Negative Results for COVID-19 IgG/IgM Antibody Testing After Heat-Inactivation
Source: Front Med (Lausanne). 2021 Jan 18;7:589080. doi: 10.3389/fmed.2020.589080 (PMC7849051; doi:10.3389/fmed.2020.589080)
Supplement: Supplementary file 1 [file Table_1.DOCX]

Supporting information

Table S1. The cutoff values for SARS-CoV-2-specific IgG antibody detection with indirect immunity-based kit produced by manufacturer A

|  | **Control** | **56 °C for 30 min** | **56 °C for 45 min** | **56 °C for 60 min** | **60 °C for 30 min** | **65 °C for 30 min** |
| --- | --- | --- | --- | --- | --- | --- |
|  | 70.69 | 105.52 | 253.42 | 252.52 | 185.34 | 235.21 |
|  | 96.6 | 96.79 | 254.57 | 261.32 | 233.95 | 214.98 |
|  | 83.82 | 117.29 | 244.73 | 240.16 | 165.82 | 244.67 |
|  | 66.16 | 133.91 | 325.26 | 302.55 | 176.02 | 316.93 |
|  | 39.97 | 44.77 | 82.49 | 79.21 | 70.34 | 71.22 |
|  | 89.63 | 93.57 | 191.4 | 207.39 | 52.79 | 202.27 |
|  | 62.81 | 71.04 | 155.13 | 147.3 | 139.37 | 147.51 |
|  | 0.87 | 1.03 | 3.23 | 7.44 | 186.41 | 3.05 |
|  | 44.72 | 105.62 | 211.3 | 216.53 | 136.32 | 189.31 |
|  | 73.11 | 4.44 | 281.27 | 290.44 | 201.85 | 276.7 |
|  | 60.16 | 62.88 | 142.97 | 136.47 | 168.52 | 134.94 |
|  | 60.59 | 63.96 | 140.42 | 138.57 | 121.2 | 130.48 |
|  | 0.66 | 1.32 | 8.53 | 7.96 | 2.28 | 3.06 |
|  | 87.32 | 127.71 | 270.45 | 239.82 | 143.98 | 235.29 |
|  | 47.29 | 137.04 | 274.01 | 298.57 | 216 | 210.75 |
|  | 26.65 | 26.84 | 58.46 | 47.12 | 126.67 | 56.07 |
|  | 65.81 | 69.8 | 176.59 | 182.14 | 124.99 | 165.65 |
|  | 54.2 | 138.03 | 324.03 | 369.84 | 1.69 | 271.92 |
|  | 76.65 | 82.13 | 184.24 | 170.19 | 194.88 | 148.04 |
|  | 57.94 | 121.91 | 338.23 | 335.01 | 136.12 | 242.98 |
|  | 22.48 | 30.36 | 58.83 | 58.26 | 55.89 | 59.6 |
|  | 51.26 | 69.68 | 169.02 | 180.29 | 128.96 | 136.53 |
|  | 69.56 | 77.35 | 183.77 | 153.45 | 137.86 | 66.51 |
|  | 64.35 | 65.74 | 131.29 | 131.59 | 129.35 | 64.05 |
|  | 67.11 | 125.35 | 279.53 | 291.23 | 201.15 | 113.15 |
|  | 22.44 | 22.64 | 53.87 | 63.39 | 44.16 | 25.99 |
|  | 80.83 | 112.51 | 287.4 | 266.87 | 174.31 | 108.75 |
|  | 59.61 | 124.55 | 283.18 | 256.75 | 171.09 | 95.01 |
|  | 21.46 | 22.3 | 48.16 | 47.87 | 41.24 | 22.38 |
|  | 90.32 | 126.98 | 294.18 | 267.89 | 210.31 | 117.74 |
|  | 41.46 | 50.9 | 106.7 | 100.64 | 78.91 | 46.35 |
|  | 60.74 | 99.44 | 281.53 | 218.22 | 174.8 | 84.5 |
|  | 73.92 | 77.86 | 208.1 | 222.49 | 189.04 | 79.93 |
|  | 67.29 | 72.8 | 144.28 | 158.69 | 133.34 | 69.04 |
|  | 2.61 | 3.97 | 9.81 | 10 | 5.72 | 2.78 |
|  | 81.78 | 95.13 | 154.11 | 179.4 | 163.17 | 83.7 |
|  | 83.35 | 86.78 | 173.5 | 190.67 | 175.6 | 79.77 |
|  | 48.16 | 124.74 | 317.9 | 302.99 | 151.03 | 72.82 |
|  | 95.23 | 98.85 | 229.63 | 208.25 | 174.92 | 97.92 |
|  | 88.48 | 116.71 | 274.12 | 243.88 | 213.95 | 112.62 |
|  | 44.05 | 103.28 | 245.81 | 252.96 | 140.27 | 78.57 |
|  | 23.73 | 25.55 | 55.29 | 47.7 | 40.22 | 23.53 |
|  | 48.99 | 137.74 | 303.34 | 309.55 | 137.86 | 88.6 |
|  | 53.14 | 141.15 | 307.03 | 290.08 | 151.38 | 99.48 |
|  | 34.27 | 88.81 | 182.94 | 183.85 | 79.24 | 49.94 |
|  | 69.9 | 97.77 | 229.09 | 219.07 | 159.65 | 91.65 |
|  | 35.85 | 36.99 | 74.53 | 79.03 | 71.39 | 36 |
|  | 74.65 | 79.39 | 159.92 | 159.12 | 129.14 | 130.01 |
|  | 83.71 | 85.31 | 183.14 | 188.53 | 175.09 | 151.85 |
|  | 80.33 | 120.92 | 275.65 | 269.81 | 177.22 | 203.96 |
|  | 60.17 | 152.13 | 320.06 | 300.82 | 164.62 | 233.36 |
|  | 68.13 | 81.86 | 157.45 | 128.31 | 119.11 | 141.49 |
|  | 92.3 | 116.07 | 259.9 | 261.82 | 212.58 | 242.31 |
|  | 39.34 | 367.34 | 360.06 | 154.92 | 307.96 | 0.77 |
|  | 69.13 | 174.63 | 205.47 | 95.48 | 157.92 | 161.98 |
|  | 41.84 | 98.52 | 71.38 | 42.49 | 71.02 | 0.13 |
|  | 71.91 | 268.98 | 228.93 | 103.35 | 215.29 | 218.86 |
|  | 64.91 | 207.44 | 215.01 | 97.11 | 194.27 | 185.46 |
|  | 85.31 | 264.2 | 274.74 | 101.95 | 247.06 | 283.59 |
|  | 54.02 | 94.46 | 55.33 | 52.43 | 85.16 | 80.15 |
|  | 67.85 | 282.8 | 118.4 | 119.82 | 201.78 | 208.43 |
|  | 62.74 | 284.36 | 119.57 | 112.34 | 232.93 | 238.99 |
|  | 11.58 | 32.59 | 13.77 | 13.76 | 20.32 | 0.31 |
|  | 69.1 | 260.46 | 117.3 | 108.72 | 256.21 | 267.47 |
|  | 61.74 | 321.45 | 135.06 | 116.41 | 248.52 | 310.29 |
|  | 60.95 | 171.1 | 86.11 | 85.25 | 162.13 | 169.14 |
|  | 43.14 | 234.87 | 122.54 | 129.85 | 245.13 | 250.99 |
|  | 58.23 | 295.98 | 127.1 | 125.23 | 249.57 | 270.93 |
|  | 86.11 | 233.54 | 102.34 | 103.44 | 193.26 | 207.07 |
|  | 23.01 | 48.44 | 17.94 | 17.43 | 38.82 | 37.7 |
|  | 69.06 | 270.85 | 111.67 | 116.97 | 232.01 | 261.06 |
|  | 50.16 | 102.62 | 49.69 | 44.81 | 84.54 | 144.24 |
|  | 74.17 | 162.96 | 71.88 | 72.16 | 100.09 | 132.47 |
|  | 76.03 | 230.8 | 92.56 | 115.49 | 272.24 | 250.09 |
|  | 66.35 | 220.47 | 90.48 | 92.46 | 241.11 | 232.77 |
|  | 46.81 | 165.17 | 83.63 | 89.47 | 149.91 | 149.55 |
|  | 81.91 | 273.22 | 122.36 | 115.66 | 273.87 | 271.62 |
|  | 38.08 | 68.19 | 37.42 | 31.43 | 78.12 | 62.94 |
|  | 80.43 | 242.44 | 123 | 106.59 | 260.84 | 266.19 |
|  | 59.38 | 135.7 | 62.99 | 55.6 | 133.22 | 121.37 |
|  | 67.51 | 148.33 | 69.14 | 59.87 | 140.27 | 111.23 |
|  | 41.87 | 167.48 | 78.52 | 77.43 | 163.81 | 145.51 |
|  | 73.25 | 285.9 | 131.08 | 140.2 | 280.25 | 238.92 |
|  | 74.41 | 246.2 | 111.84 | 112.37 | 249.09 | 245.85 |
|  | 68.72 | 154.84 | 60.87 | 75.09 | 204.36 | 211.06 |
|  | 63.42 | 221.37 | 101.05 | 89.55 | 240.03 | 172.64 |
|  | 81.61 | 159.29 | 83.27 | 81.76 | 166.34 | 150.32 |
|  | 185.71 | 240.15 | 106.61 | 102.02 | 226.65 | 204.52 |
|  | 190.84 | 278.67 | 124.48 | 129.12 | 290.6 | 271.7 |
|  | 177.86 | 236.06 | 107.83 | 116.74 | 268 | 248.04 |
|  | 197.56 | 252.52 | 102.31 | 104.27 | 281.9 | 291.55 |
|  | 137.16 | 253.84 | 119.76 | 120.95 | 286.39 | 274.83 |
|  | 67.29 | 229.73 | 273.28 | 115.91 | 188.98 | 254.63 |
|  | 53.94 | 174.28 | 186.48 | 76.22 | 161.01 | 142.51 |
|  | 0.51 | 1.97 | 2.37 | 0.82 | 1.86 | 8.02 |
|  | 50.87 | 234.37 | 240.01 | 103.22 | 213.1 | 240.07 |
|  | 86.87 | 193.97 | 155.65 | 85.8 | 142.96 | 142.9 |
|  | 42.89 | 294.94 | 309.08 | 122.51 | 281.12 | 341.56 |
|  | 68.22 | 252.52 | 121.65 | 119.72 | 242.15 | 267.43 |
|  | 56.82 | 116.08 | 48.32 | 48.36 | 104.36 | 114.97 |
|  | 79.07 | 246.32 | 112.12 | 113.82 | 217.66 | 242.39 |
|  | 60.87 | 125.89 | 56.13 | 55.86 | 91.24 | 0.12 |
|  | 76.95 | 184.9 | 75.79 | 83.53 | 134.64 | 163.54 |
|  | 85.93 | 237.75 | 121.57 | 111.89 | 227.57 | 209.42 |
|  | 73.93 | 309.22 | 128.67 | 114.8 | 214.37 | 291.24 |
|  | 43.7 | 232.88 | 115.57 | 104.92 | 223.66 | 268.04 |
|  | 71.87 | 264.98 | 113.76 | 108.62 | 203.48 | 247.39 |
|  | 54.95 | 166.32 | 73.27 | 85.44 | 170.2 | 209.61 |
|  | 67.76 | 213.93 | 89.25 | 91.45 | 222.15 | 309.96 |
|  | 60.83 | 184.64 | 78.41 | 88.69 | 174.28 | 275.28 |
|  | 77.6 | 227.04 | 115.22 | 117.66 | 213.01 | 221.85 |
|  | 56.69 | 342.18 | 138.36 | 125.53 | 297.95 | 322.01 |
|  | 90.46 | 206.21 | 88.11 | 95.61 | 175.9 | 192.8 |
|  | 77.25 | 170.47 | 59.88 | 80.28 | 140.54 | 150.43 |
|  | 14.21 | 24.07 | 11.7 | 13.32 | 24.66 | 24.11 |
|  | 87.27 | 192.73 | 93.95 | 79.92 | 189.65 | 165.93 |
|  | 67.26 | 233.56 | 111.57 | 107.41 | 229.79 | 244.03 |
|  | 28.29 | 60.58 | 29.32 | 29.21 | 56.2 | 46.4 |
|  | 63.08 | 349.36 | 144.21 | 145.4 | 336.25 | 350.97 |
|  | 47.65 | 102.54 | 42.8 | 49.51 | 101.23 | 88.52 |
|  | 84.93 | 242.78 | 123.77 | 123.64 | 265.18 | 257.68 |
|  | 114.25 | 350.66 | 150.04 | 139.9 | 353.75 | 295.31 |
|  | 108.53 | 242.05 | 109.54 | 113.52 | 237.1 | 233.54 |
|  | 63.16 | 207.54 | 84.16 | 95.09 | 234 | 207.77 |
|  | 56.76 | 304.13 | 139.91 | 149.44 | 350.57 | 304.29 |
|  | 67.89 | 191.31 | 79.04 | 80.49 | 254.45 | 316.38 |
|  | 161.89 | 195.13 | 85.09 | 98.5 | 219.14 | 193.47 |
|  | 163.93 | 325.81 | 129.42 | 131.77 | 299.17 | 265.73 |
|  | 120.72 | 297.56 | 123.68 | 129.98 | 294.32 | 281.99 |

Table S2. The cutoff values for SARS-CoV-2-specific IgM antibody detection with indirect immunity-based kit produced by manufacturer A

|  | **Control** | **56 °C for 30 min** | **56 °C for 45 min** | **56 °C for 60 min** | **60 °C for 30 min** | **65 °C for 30 min** |
| --- | --- | --- | --- | --- | --- | --- |
|  | 13.71 | 7.34 | 19.61 | 20.33 | 7.05 | 12.64 |
|  | 8.53 | 4.35 | 19.59 | 18.68 | 2.21 | 2.27 |
|  | 11.97 | 4.27 | 11.31 | 10.99 | 4.3 | 4.44 |
|  | 29.72 | 9.97 | 23.98 | 24.67 | 7.72 | 6.91 |
|  | 23.58 | 12.59 | 25.2 | 24.05 | 13.28 | 12.65 |
|  | 44.27 | 11.85 | 28.11 | 30.73 | 8.22 | 13.95 |
|  | 27.3 | 5.32 | 14.71 | 14.83 | 2.56 | 9.05 |
|  | 18.56 | 6.91 | 14.07 | 13.32 | 38.89 | 8.42 |
|  | 56 | 14.38 | 32.75 | 31.84 | 8.53 | 14.84 |
|  | 16.26 | 6.47 | 14.77 | 13.77 | 5.4 | 7.09 |
|  | 16.47 | 6.84 | 17.69 | 17.76 | 15.72 | 3.78 |
|  | 25.78 | 5.97 | 16.51 | 15.29 | 9.33 | 5.56 |
|  | 1.14 | 0.52 | 3.04 | 3.14 | 9.4 | 2.81 |
|  | 101.43 | 15.33 | 28.66 | 25.23 | 14.8 | 44.72 |
|  | 116.2 | 44.05 | 97.52 | 94.54 | 7.48 | 38.28 |
|  | 14.41 | 7.05 | 19.83 | 14.63 | 3.88 | 8.9 |
|  | 5.7 | 2.59 | 9.01 | 7.67 | 6.27 | 2.76 |
|  | 72.67 | 35.25 | 83.19 | 76.49 | 0.78 | 40.05 |
|  | 36.36 | 6.47 | 16.19 | 14.62 | 46.85 | 10 |
|  | 11.06 | 7.72 | 21.52 | 21.62 | 41.26 | 5.67 |
|  | 6.73 | 2.5 | 13.76 | 12.71 | 3.62 | 3.02 |
|  | 119.11 | 40.55 | 90.82 | 84.85 | 40.92 | 40.98 |
|  | 81.53 | 20.38 | 39.33 | 36.79 | 28.93 | 26.99 |
|  | 5.67 | 2.67 | 5.69 | 5.42 | 3.6 | 3.29 |
|  | 43.17 | 17.14 | 44.08 | 38.92 | 14.57 | 12.3 |
|  | 2.15 | 1.16 | 4.61 | 4.49 | 1.61 | 1.34 |
|  | 7.85 | 3.12 | 7.99 | 8.26 | 3.74 | 3.14 |
|  | 43.73 | 19.55 | 56.92 | 54.13 | 15.26 | 15.24 |
|  | 2.32 | 1.6 | 5.28 | 4.63 | 1.53 | 1.29 |
|  | 1.99 | 1.01 | 4.04 | 3.96 | 0.95 | 2.06 |
|  | 71.67 | 22.79 | 50.35 | 44.57 | 28.85 | 32.11 |
|  | 96.76 | 30.71 | 58.62 | 53.22 | 36.82 | 37.07 |
|  | 4.58 | 2.22 | 5.72 | 5.99 | 2.3 | 4.34 |
|  | 18.08 | 3.5 | 8.47 | 9.37 | 5.2 | 9.23 |
|  | 0.67 | 0.59 | 1.49 | 1.63 | 0.65 | 0.79 |
|  | 3.4 | 1.94 | 6.03 | 4.64 | 1.84 | 1.99 |
|  | 54.99 | 19.37 | 41.63 | 37.27 | 26.55 | 29.4 |
|  | 121.63 | 35.59 | 73.17 | 80.15 | 43.66 | 42.65 |
|  | 46.09 | 12.05 | 25.69 | 27.58 | 17.01 | 17.15 |
|  | 7.04 | 2.24 | 6.07 | 6.37 | 3.59 | 3.41 |
|  | 117.33 | 30.14 | 69.25 | 74.02 | 40.62 | 33.88 |
|  | 4.51 | 3.44 | 7.71 | 10.06 | 2.21 | 3.23 |
|  | 6.71 | 6.89 | 14.26 | 16.13 | 9.34 | 8.64 |
|  | 51.32 | 41.1 | 95.09 | 108.33 | 39.57 | 38.75 |
|  | 2.83 | 6.55 | 21.11 | 34.36 | 2.72 | 2.74 |
|  | 9.81 | 10.53 | 25.69 | 30.77 | 7.48 | 5.53 |
|  | 1 | 1.46 | 5.01 | 6.49 | 1.03 | 1.17 |
|  | 40.35 | 30.47 | 66.86 | 58.12 | 95.31 | 34.88 |
|  | 6.22 | 5.09 | 12.31 | 9.43 | 14.26 | 5.95 |
|  | 12.77 | 11.17 | 30.74 | 30.85 | 29.57 | 10.3 |
|  | 10.12 | 10.93 | 23.7 | 25.91 | 21.43 | 10.19 |
|  | 5.47 | 4.96 | 9.31 | 8.59 | 13 | 4.93 |
|  | 17.52 | 15.68 | 42.61 | 47.12 | 68.6 | 17.15 |
|  | 4.06 | 9.39 | 5.34 | 5.55 | 10.76 | 1.06 |
|  | 30.67 | 50.79 | 14.48 | 9.67 | 24.07 | 16.15 |
|  | 5.87 | 8.37 | 2.51 | 1.7 | 2.23 | 1.44 |
|  | 14.07 | 35.23 | 14.83 | 14.63 | 22.19 | 14.61 |
|  | 11.62 | 41.53 | 20.91 | 20.27 | 32.3 | 20.47 |
|  | 18.83 | 28.69 | 11.66 | 10 | 16.22 | 14.36 |
|  | 21.42 | 30.32 | 8.99 | 6.68 | 10.42 | 3.81 |
|  | 7.2 | 19.27 | 8.42 | 8.61 | 14.23 | 19.88 |
|  | 25.06 | 46.06 | 22.03 | 21.56 | 32.92 | 75.37 |
|  | 1.28 | 4.59 | 2.63 | 2.4 | 3.25 | 0.51 |
|  | 22.58 | 33.77 | 15.67 | 13.07 | 19.03 | 8.33 |
|  | 25.53 | 52.83 | 25.11 | 24.38 | 32.59 | 24.24 |
|  | 38.09 | 83.95 | 38.63 | 33.39 | 14.42 | 8.85 |
|  | 24.04 | 28.05 | 11 | 10.52 | 12.69 | 6.22 |
|  | 9.27 | 42.93 | 20.64 | 21.19 | 30.15 | 23.17 |
|  | 6.54 | 10.41 | 3.63 | 3.12 | 7.44 | 4.16 |
|  | 1.04 | 5.53 | 1.47 | 1.74 | 14.27 | 2.75 |
|  | 24.93 | 32.63 | 13.4 | 11.55 | 23.39 | 10.43 |
|  | 1.64 | 3.41 | 1.35 | 0.95 | 2.18 | 113.5 |
|  | 3 | 9.89 | 4.04 | 3.98 | 7.65 | 8.08 |
|  | 22.69 | 40.51 | 15.34 | 15.43 | 35.36 | 11.11 |
|  | 30.9 | 42.92 | 18.01 | 16.82 | 32.85 | 15.58 |
|  | 36.88 | 71.78 | 32.45 | 30.62 | 59.44 | 32.83 |
|  | 1.86 | 10.65 | 10.79 | 7.48 | 21.18 | 24.7 |
|  | 5.09 | 11.09 | 5.03 | 5.06 | 7.72 | 4.55 |
|  | 13.52 | 29.6 | 13.17 | 13.6 | 24.65 | 32.57 |
|  | 6.91 | 10.86 | 4.99 | 5.04 | 6.01 | 3.91 |
|  | 5.92 | 11.55 | 5.65 | 5.26 | 7.33 | 5.75 |
|  | 49.72 | 62.41 | 29.19 | 30.27 | 19.52 | 5.09 |
|  | 54.14 | 36.97 | 16.14 | 17.97 | 39.99 | 15.85 |
|  | 16.44 | 16.81 | 7.83 | 8.44 | 15.73 | 32.51 |
|  | 10.48 | 10.38 | 3.75 | 4.54 | 10.24 | 8.51 |
|  | 77.54 | 50.72 | 23.11 | 20.92 | 33.7 | 17.82 |
|  | 10.22 | 13.69 | 4.4 | 4.85 | 19 | 17.36 |
|  | 8.85 | 17 | 10.05 | 10.67 | 38.49 | 32.26 |
|  | 48.41 | 36.43 | 16.34 | 15.09 | 32.84 | 11.59 |
|  | 22.33 | 16.35 | 8.17 | 7.93 | 15.92 | 32.51 |
|  | 54.6 | 33.25 | 11.54 | 11.89 | 20.14 | 14.59 |
|  | 11.8 | 16.91 | 8.14 | 8.04 | 31.21 | 21.65 |
|  | 11.49 | 16.7 | 7.26 | 7.53 | 13.53 | 38.52 |
|  | 25.96 | 45.35 | 16.98 | 14.99 | 21.36 | 13.02 |
|  | 4.69 | 2.21 | 2 | 0.86 | 2.15 | 5.06 |
|  | 79.43 | 145.22 | 56.11 | 51.95 | 63.87 | 20.07 |
|  | 1.85 | 3.72 | 1.95 | 3.38 | 5.81 | 12.71 |
|  | 18.56 | 42.32 | 17.97 | 19.73 | 130.89 | 38.28 |
|  | 2.5 | 4.21 | 1.63 | 1.54 | 30.2 | 14.73 |
|  | 6.13 | 19.15 | 9.87 | 10.48 | 4.2 | 4.92 |
|  | 1.45 | 4.15 | 2.19 | 2.47 | 17.94 | 22.57 |
|  | 2.78 | 6.16 | 2.54 | 3.05 | 3.76 | 0.34 |
|  | 15.65 | 23.28 | 10.05 | 9.53 | 17.91 | 11.45 |
|  | 12.39 | 19.59 | 8.73 | 9.41 | 22.53 | 27.59 |
|  | 102.37 | 117.27 | 48.8 | 46.49 | 67.02 | 34.06 |
|  | 6 | 31.28 | 14.12 | 15.71 | 22.87 | 24.89 |
|  | 9.27 | 11.79 | 4.66 | 4.62 | 10.07 | 8.37 |
|  | 3.7 | 4.94 | 3 | 3.52 | 6.72 | 15.34 |
|  | 24.85 | 61.02 | 25.78 | 23.27 | 41.34 | 22.83 |
|  | 1.44 | 5.45 | 3.96 | 3.37 | 11.92 | 7.11 |
|  | 7.83 | 34.96 | 14.14 | 14.86 | 27.99 | 23.99 |
|  | 2.35 | 15.14 | 6.53 | 8.74 | 17.73 | 13.95 |
|  | 18.62 | 26.26 | 8.65 | 7.34 | 14.83 | 7.08 |
|  | 1.05 | 3.44 | 1.83 | 2.31 | 3.81 | 6.63 |
|  | 1.52 | 3.06 | 1.56 | 1.63 | 3.86 | 2.13 |
|  | 2.02 | 4.9 | 2.45 | 2.74 | 6.28 | 0.65 |
|  | 1.24 | 2.52 | 1 | 1.04 | 1.9 | 1.64 |
|  | 3.51 | 23.26 | 9.66 | 10.41 | 34.35 | 38.19 |
|  | 3.46 | 9.7 | 4.34 | 4.31 | 7.48 | 2.74 |
|  | 8.75 | 23.75 | 9.15 | 9.12 | 25.64 | 24.19 |
|  | 13.71 | 8.09 | 3.75 | 4.24 | 11.83 | 33.63 |
|  | 31.21 | 16.22 | 5.37 | 6.04 | 15.9 | 9.62 |
|  | 78.61 | 53.58 | 22.58 | 20.69 | 27.52 | 7.3 |
|  | 26.15 | 29.74 | 12.38 | 13.15 | 31.41 | 23.96 |
|  | 4.22 | 4.73 | 3.69 | 2.59 | 9.4 | 11.92 |
|  | 3.99 | 8.59 | 5.14 | 4.76 | 15.95 | 19.95 |
|  | 25.79 | 34.51 | 14.67 | 17.41 | 43.69 | 26.48 |
|  | 62.7 | 35.4 | 14.53 | 18.13 | 21.85 | 10.65 |
|  | 53.07 | 49.46 | 25.26 | 24.81 | 41.45 | 37.21 |

Table S3. The cutoff values for SARS-CoV-2-specific IgG antibody detection with indirect immunity-based kit produced by manufacturer B

|  | **Control** | **56 °C for 30 min** | **56 °C for 45 min** | **60 °C for 30 min** |
| --- | --- | --- | --- | --- |
|  | 97.09 | 90.67 | 94.7 | 78.27 |
|  | 116.08 | 109.91 | 111.12 | 97.61 |
|  | 154.2 | 158.44 | 164.32 | 127.92 |
|  | 130.91 | 119.31 | 121.56 | 100.12 |
|  | 293.87 | 304.74 | 320.32 | 241.48 |
|  | 215.2 | 281.85 | 274.72 | 240.07 |
|  | 68.71 | 71.85 | 67.78 | 65.45 |
|  | 247.9 | 280.02 | 281.93 | 212.77 |
|  | 615.9 | 704.98 | 651.99 | 356.89 |
|  | 501.97 | 486.4 | 580.76 | 318.48 |
|  | 457.22 | 471.95 | 477.36 | 365.93 |
|  | 53.64 | 50.81 | 53.82 | 46.98 |
|  | 163.07 | 158.57 | 165.8 | 106.37 |
|  | 295.74 | 337.12 | 307.38 | 244.74 |
|  | 112.82 | 129.91 | 125.48 | 100.48 |
|  | 330.35 | 395.39 | 382.61 | 267.65 |
|  | 223.18 | 237.83 | 216.5 | 182.69 |
|  | 63.5 | 63.56 | 61.73 | 56.78 |
|  | 267.52 | 265.1 | 238.47 | 232.55 |
|  | 151.93 | 173.28 | 153.49 | 147.73 |

Table S4. The cutoff values for SARS-CoV-2-specific IgM antibody detection with indirect immunity-based kit produced by manufacturer B

|  | **Control** | **56 °C for 30 min** | **56 °C for 45 min** | **60 °C for 30 min** |
| --- | --- | --- | --- | --- |
|  | 1.85 | 1.64 | 1.33 | 0.66 |
|  | 0.89 | 0.83 | 0.79 | 0.65 |
|  | 0.71 | 0.66 | 0.58 | 0.43 |
|  | 0.41 | 0.41 | 0.42 | 0.35 |
|  | 0.71 | 0.65 | 0.61 | 0.46 |
|  | 3.23 | 2.84 | 2.86 | 1.7 |
|  | 0.86 | 0.75 | 0.76 | 0.55 |
|  | 1.11 | 1.09 | 1.1 | 0.98 |
|  | 1.58 | 1.21 | 1.16 | 0.54 |
|  | 0.74 | 0.66 | 0.63 | 0.49 |
|  | 0.38 | 0.35 | 0.32 | 0.23 |
|  | 0.52 | 0.49 | 0.46 | 0.33 |
|  | 1.5 | 1.32 | 1.32 | 0.9 |
|  | 0.42 | 0.38 | 0.35 | 0.23 |
|  | 0.92 | 0.87 | 0.84 | 0.65 |
|  | 2.19 | 2.08 | 2.02 | 1.32 |
|  | 0.91 | 0.75 | 0.69 | 0.39 |
|  | 3.82 | 1.76 | 1.49 | 0.8 |
|  | 0.33 | 0.23 | 0.19 | 0.15 |
|  | 0.74 | 0.65 | 0.58 | 0.45 |

Table S5. The cutoff values for SARS-CoV-2-specific IgM antibody detection with capture method-based kit produced by manufacturer C

|  | **Control** | **56 °C for 30 min** | **60 °C for 30 min** |
| --- | --- | --- | --- |
|  | 0.22 | 0.16 | 0.17 |
|  | 0.2 | 0.17 | 0.17 |
|  | 0.79 | 0.69 | 0.7 |
|  | 0.64 | 0.62 | 0.72 |
|  | 0.51 | 0.47 | 0.43 |
|  | 0.12 | 0.11 | 0.12 |
|  | 0.9 | 0.81 | 0.84 |
|  | 0.62 | 0.59 | 0.66 |
|  | 0.2 | 0.16 | 0.17 |
|  | 0.15 | 0.15 | 0.13 |
|  | 0.68 | 0.66 | 0.69 |
|  | 0.46 | 0.48 | 0.42 |
|  | 0.85 | 0.75 | 0.86 |
|  | 0.79 | 0.74 | 0.91 |
|  | 2.67 | 3.04 | 3.08 |
|  | 1.01 | 1.01 | 1.13 |
|  | 0.13 | 0.09 | 0.1 |
|  | 0.14 | 0.14 | 0.14 |
|  | 7.3 | 8.13 | 7.56 |
|  | 8.83 | 10.03 | 10.06 |
|  | 4.5 | 4.52 | 4.41 |
|  | 0.04 | 0.04 | 0.04 |
|  | 2.82 | 2.96 | 2.54 |
|  | 3.06 | 3.19 | 3.04 |
|  | 0.76 | 0.88 | 0.88 |
|  | 2.6 | 2.52 | 2.35 |
|  | 3.73 | 3.57 | 3.78 |
|  | 2.17 | 2.19 | 2.13 |
|  | 2.54 | 2.12 | 1.52 |
|  | 10.23 | 9.81 | 9.77 |
|  | 13.83 | 10.46 | 9.69 |
|  | 13.87 | 16.69 | 15.58 |
|  | 3.01 | 3.11 | 2.39 |
|  | 0.18 | 0.16 | 0.15 |

Table S6. The cutoff values for SARS-CoV-2 total antibody detection with double-antigen sandwich method-based kit produced by manufacturer C

|  | **Control** | **56 °C for 30 min** | **60 °C for 30 min** |
| --- | --- | --- | --- |
|  | 256.46 | 261.66 | 289.81 |
|  | 248.23 | 262.22 | 268.27 |
|  | 966.95 | 914.31 | 741.84 |
|  | 1085.06 | 967.31 | 890.2 |
|  | 518.14 | 482.07 | 406.06 |
|  | 342.4 | 343.31 | 334.73 |
|  | 1242.08 | 1166.61 | 1066.15 |
|  | 1085.87 | 999.27 | 833.24 |
|  | 244.79 | 258.65 | 270.09 |
|  | 700.86 | 658.58 | 591.7 |
|  | 904.58 | 890.16 | 748.9 |
|  | 513.67 | 490.17 | 393.42 |
|  | 1226.36 | 1241.75 | 1082.1 |
|  | 1120.86 | 1124.57 | 1034.63 |
|  | 1182.5 | 1383.6 | 1383.85 |
|  | 225.71 | 223.46 | 202.17 |
|  | 0.05 | 0.05 | 0.05 |
|  | 125.96 | 118.98 | 108.55 |
|  | 571.7 | 488.94 | 464.31 |
|  | 1194.73 | 1317.71 | 1286.18 |
|  | 157.48 | 171.67 | 177.1 |
|  | 0.08 | 0.06 | 0.05 |
|  | 819.32 | 770.38 | 694.4 |
|  | 696.12 | 718.01 | 639.09 |
|  | 218.11 | 209.54 | 169.42 |
|  | 943.82 | 858.29 | 898.34 |
|  | 124.76 | 120.01 | 85.57 |
|  | 330.54 | 306.49 | 288.04 |
|  | 1059.09 | 1149.16 | 1014.04 |
|  | 472.53 | 511.2 | 469.73 |
|  | 1131.81 | 1145.6 | 1044.96 |
|  | 542.29 | 564.29 | 504.72 |
|  | 1760.66 | 1669.63 | 1426.78 |
|  | 78.3 | 72.06 | 53.71 |

Table S7. The cutoff values for SARS-CoV-2 total antibody detection with double-antigen sandwich method-based kit produced by manufacturer D

|  | **Control** | **56 °C for 30 min** | **60 °C for 30 min** |
| --- | --- | --- | --- |
|  | 6.18 | 5.54 | 5.02 |
|  | 4.1 | 3.68 | 3.47 |
|  | 2.29 | 2.33 | 2.15 |
|  | 3.81 | 3.66 | 3.98 |
|  | 6.35 | 5.26 | 4.9 |
|  | 7.02 | 7.2 | 5.71 |
|  | 3.41 | 3.23 | 2.42 |
|  | 11.14 | 11.06 | 10.14 |
|  | 5.18 | 5.6 | 5.2 |
|  | 5.88 | 7.98 | 6.17 |
